# Associations of linoleic acid with markers of glucose metabolism and liver function in South African adults

**DOI:** 10.1186/s12944-020-01318-3

**Published:** 2020-06-16

**Authors:** Kamalita Pertiwi, Leanne K. Küpers, Johanna M. Geleijnse, Peter L. Zock, Anne J. Wanders, Herculina S. Kruger, Tertia van Zyl, Iolanthé M. Kruger, Cornelius M. Smuts

**Affiliations:** 1 Division of Human Nutrition and Health, Wageningen University, Wageningen, The Netherlands; 2 Unilever R&D, Foods Innovation Centre, Wageningen, the Netherlands; 3 Centre of Excellence for Nutrition, Faculty of Health Sciences, North-West University, Potchefstroom, South Africa; 4 Africa Unit for Transdisciplinary Health Research, Faculty of Health Sciences, North-West University, Potchefstroom, South Africa

**Keywords:** Linoleic acid, Polyunsaturated fatty acids, Gamma-glutamyl transferase, Liver enzymes, Glycemia, African, Alcohol intake

## Abstract

**Background:**

The relation between dietary and circulating linoleic acid (18:2 n-6, LA), glucose metabolism and liver function is not yet clear. Associations of dietary and circulating LA with glucose metabolism and liver function markers were investigated.

**Methods:**

Cross-sectional analyses in 633 black South Africans (aged > 30 years, 62% female, 51% urban) without type 2 diabetes at baseline of the Prospective Urban Rural Epidemiology study. A cultural-sensitive 145-item food-frequency questionnaire was used to collect dietary data, including LA (percentage of energy; en%). Blood samples were collected to measure circulating LA (% total fatty acids (FA); plasma phospholipids), plasma glucose, glycosylated hemoglobin (HbA1c), serum gamma-glutamyl transferase (GGT), alanine (ALT) and aspartate aminotransferase (AST). Associations per 1 standard deviation (SD) and in tertiles were analyzed using multivariable regression.

**Results:**

Mean (±SD) dietary and circulating LA was 6.8 (±3.1) en% and 16.0 (±3.5) % total FA, respectively. Dietary and circulating LA were not associated with plasma glucose or HbA1c (β per 1 SD: − 0.005 to 0.010, *P* > 0.20). Higher dietary LA was generally associated with lower serum liver enzymes levels. One SD higher circulating LA was associated with 22% lower serum GGT (β (95% confidence interval): − 0.25 (− 0.31, − 0.18), *P* < 0.001), but only ≤9% lower for ALT and AST. Circulating LA and serum GGT associations differed by alcohol use and locality.

**Conclusion:**

Dietary and circulating LA were inversely associated with markers of impaired liver function, but not with glucose metabolism. Alcohol use may play a role in the association between LA and liver function.

**Trial registration:**

PURE North-West Province South Africa study described in this manuscript is part of the PURE study. The PURE study is registered in ClinicalTrials.gov (Identifier: NCT03225586; URL).

## Introduction

Circulating linoleic acid (18:2 n-6, LA) has been inversely associated with the risk of type 2 diabetes (T2D) in cohorts of mostly healthy Caucasian populations [1], whereas the association between dietary linoleic acid and T2D risk remains equivocal [2–5]. This discrepancy has been mainly attributed to the limitation of self-reported dietary LA assessment. However, endogenous fatty acids (FA) metabolism affecting circulating LA proportion may also play a role.

The proportion of circulating LA is not only influenced by dietary LA, but also by endogenous FA metabolism, which may be affected by various factors such as obesity and liver impairment [6, 7]. A lower circulating LA was observed in non-alcoholic fatty liver disease patients as compared to healthy individuals [8]. Circulating LA has also been inversely associated with hepatic fat deposition [9]. These findings suggest that low proportions of circulating LA may indicate impairment of liver function.

The influence of dietary LA on glucose metabolism and liver function is not yet clear. Meta-analyses of randomized controlled feeding trials showed that increasing the intake of polyunsaturated fatty acids (PUFA) (mainly LA) potentially affects glucose metabolism [10, 11]. On the other hand, a high dietary n-6 FA or n-6/n-3 ratio has been considered to promote the development of fatty liver through inflammation [12], although evidence from two systematic reviews and meta-analyses of randomized controlled trials in humans showed that increasing dietary n-6 FA did not result in higher concentration of inflammatory markers [13, 14].

Studies on LA and markers related to T2D risk have been performed mainly in Caucasian populations. Populations with African ancestry may have different distributions of genetic variants related to long-chain PUFA metabolism [15]. Therefore, this study aimed to investigate associations of both dietary and circulating LA with markers of glucose metabolism and liver function in the South African leg of the Prospective Urban and Rural Epidemiological (PURE) study.

## Subjects and methods

### Study population

The PURE North-West Province South Africa (PURE-NWP-SA) is a cohort of black South African participants and part of the larger international PURE study. The design, selection and recruitment of participants for the PURE-NWP-SA study have been described previously [16]. In brief, apparently healthy black South African adults aged > 30 years old were recruited from two urban and two rural communities in North-West Province. Of 3750 adults who completed the screening questionnaire, 2010 attended baseline measurements in 2005. All participants gave written consent to participation and the study was approved by the Ethics Committee of the North-West University (04 M10 and NW-00016-10-A1) [16].

Baseline PURE-NWP-SA cohort data were used for the present cross-sectional study. Participants without information on dietary LA intake or with implausible energy intake (< 3347 kJ (kJ) [< 800 kcal (kcal)] or > 16,736 kJ [> 4000 kcal] for men; < 2092 kJ [< 500 kcal] or > 14,644 kJ [> 3500 kcal] for women) [17] or missing plasma FA were further excluded. Missing or unreliable plasma glucose (values < 2.5 mmol/L) and glycosylated hemoglobin (HbA1c) were excluded. Finally, participants with prevalent diabetes, defined as self-reported diagnosis of type 2 diabetes or use of anti-diabetes drugs, were excluded. A total of 633 participants were included in the present analyses (Additional file 1**: Figure S1**).

### Dietary assessment

Information on usual diet was collected with a cultural-sensitive 145-item quantified food-frequency questionnaire (QFFQ). This questionnaire, covering dietary intake in the previous month, was developed and validated in a similar population [18–20]. Dietary FA intakes at baseline (expressed as grams/day (g/d) and a percentage of energy (en%)) were calculated by using the South African food composition database [21]. In addition, information from the QFFQ and food composition database were used to calculate total daily intakes of energy (kJ), macronutrients (en%), dietary fiber (g/d) and cholesterol (mg/d).

### Circulating plasma phospholipid FA measurement

Venous blood samples were collected from participants after 8–10 h of fasting by a trained nurse, prepared in conditions required for further processing and stored at − 80 °C until analysis [16]. Before analysis, frozen EDTA samples were thawed and total lipids were extracted by using a modified Folch method [22] from the plasma samples. Phospholipids FA fraction was isolated by using thin-layer chromatography. FA composition of plasma phospholipids was analyzed using quadrupole gas chromatography electron ionization mass spectrometry at the Centre of Excellence for Nutrition laboratory, Potchefstroom, South Africa as previously described in detail [23]. A total of 26 FA were quantified as a percentage of total FA identified in the sample (g/100 g total FA; % total FA) including LA and arachidonic acid (20:4n-6). Delta-6-desaturase activity was also estimated by calculating the ratio of circulating dihomo-gamma-linolenic acid to LA (20:3n-6/18:2n-6).

### Markers of glucose metabolism and liver function

Plasma glucose (mmol/L) in the fasting state was measured by using Synchron® System (Beckman Coulter Co., Fullerton, CA, USA). HbA1c was assessed in EDTA-treated whole blood by using a D-10 Hemoglobin testing system (Bio-Rad Laboratories, Hercules, CA, USA). Serum liver enzymes (gamma-glutamyl transferase (GGT), alanine aminotransferase (ALT), aspartate aminotransferase (AST); U/L) were determined using Sequential Multiple Analyzer Computer Konelab20i auto analyzer (Thermo Fischer Scientific, Vantaa, Finland).

### Other measurements

Information about demographics, socioeconomic status, lifestyle and physical activity was collected using structured questionnaires that were standardized for the international PURE study [16]. Locality was identified as rural or urban based on the communities where the participants resided. Physical activity index (PAI) was calculated (maximum score of 10.0) and participants were categorized into having ‘low’(1 < PAI ≤ 3.33), ‘moderate’ (3.34 ≤ PAI ≤ 6.67), or ‘high’ (PAI ≥6.68) physical activity [24, 25]. Participants had to indicate their level of education obtained and their responses were recoded into a dichotomous variable of ‘no school or primary’ or ‘secondary or higher’. Participants’ smoking status was recorded as ‘non-smoker’ or ‘past or current smoker’. Alcohol intake was assessed by using the QFFQ and expressed as g/d of pure ethanol [26]. Alcohol intake was also categorized into ‘no’(0 g/d), ‘light’(> 0–10 g/d (women); > 0–20 g/d (men)), ‘moderate’ (> 10–20 g/d (women); > 20–30 g/d (men)), or ‘high’(> 20 g/d (women); > 30 g/d (men)).

Height, weight and waist circumference were measured using standardized procedure as described previously [16]. Body mass index (BMI) was calculated by dividing weight by height squared (kg/m^2^). Obesity was considered present when BMI ≥30 kg/m^2^. Other biochemical analyses such as total cholesterol (mmol/L), high-density lipoprotein cholesterol (HDL-C; mmol/L), triglycerides (TG; mmol/L), high-sensitivity C-reactive protein (mg/L) were determined in serum [27]. Interleukin-6 (pg/ml) was measured using ultrasensitive enzyme immunoassays (Elecsys 2010, Roche, Basel, Switzerland) [28]. Low-density lipoprotein cholesterol (LDL-C; mmol/L) was estimated using the equation: LDL-C = total cholesterol − HDL-C − triglycerides/adjustable factor. In this modification of the Friedewald equation specific adjustable factors are used to estimate VLDL-C, depending on the levels of triglycerides and non-HDL-C [29]. Values of serum HDL-C ≤ 1.0 mmol/L for men or ≤ 1.3 mmol/L for women were considered low and serum triglycerides > 1.7 mmol/L was considered as high. Systolic and diastolic blood pressure (in mmHg) were measured twice on the right upper arm in a seating position with 5 min between measurements as previously reported [27]. Use of medications (anti-diabetes, anti-hypertensive drug) was recorded at study sites by interviewers. Hypertension was considered present if blood pressure was ≥140/90 mmHg or using antihypertensive medication. The human immunodeficiency virus (HIV) status of participants was assessed according to the South African National Department of Health protocol as previously described [16].

### Statistical analysis

Participants’ characteristics were examined in the total sample and by categorizing the participants into tertiles according to their dietary or circulating LA values. Linear trends across dietary or circulating LA were calculated by assigning median values of dietary or circulating LA for the tertile and modelling these as a continuous variable in the regression models. The distribution of values for all variables, the residuals and homogeneity of variances were examined before performing the main analyses. The outcome variables (plasma glucose, HbA1c, serum GGT, ALT and AST) were log-transformed before analysis to achieve normality of residuals and homogeneity of variances.

To assess the relationship between dietary and circulating LA, Spearman rank correlation (r) between dietary and circulating LA was calculated and adjusted for age, sex and total energy intake. Least-squares means and 95% confidence interval (CI) of circulating LA (% total FA) across tertiles of dietary LA (en%) were calculated by using linear regression, adjusted for age, sex and total energy intake, also in subgroups by sex (men vs. women), age (< 65 vs. ≥65 y), obesity (present vs. absent), glycemic status (plasma glucose < 6.1 vs. ≥6.1 mmol/L), and alcohol use (‘no’: intake = 0 g/d vs. ‘yes’: intake > 0 g/d).

Association of dietary or circulating LA with glycemic and liver function measures were evaluated per 1 SD and in dietary or circulating LA tertiles by using multivariable linear regression. Model 1 included age and sex. Model 2 was additionally adjusted for education level, locality, smoking status, physical activity, alcohol intake (g/d), total energy intake (kJ/d) and BMI (kg/m^2^). Model 3 was further adjusted for available carbohydrates (en%), saturated FA (en%), trans-FA (g/d) and dietary fiber (g/d). Dietary factors were considered but not included in the fully adjusted model for circulating LA (model 2) because no indication was found for confounding of circulating LA associations by these factors. Missing values in covariables (education level, *n* = 15; smoking status, *n* = 1; physical activity, *n* = 22) were imputed by using sex-specific modes. Potential effect modification by sex, age, locality, obesity, and alcohol use was evaluated by using stratified analyses and by including product terms in the fully adjusted model (model 3 for dietary LA and model 2 for circulating LA). Stratified analyses by alcohol use were conducted in two categories (yes vs. no) due to the relatively small sizes of moderate and high alcohol intake subgroups.

In sensitivity analyses, participants with a high alcohol intake (alcohol > 20 g/d for women and > 30 g/d for men), with liver enzymes values indicating possible alcohol abuse (serum GGT > 80 U/L and AST-to-ALT ratio ≥ 2:1) or having elevated plasma glucose (fasting plasma glucose ≥6.1 mmol/L) were excluded. All analyses were performed in SAS version 9.4 (Cary, NC, USA). A two-sided *P*-value < 0.05 was considered as statistically significant.

## Results

The total sample of 633 subjects was on average 53 years, 63% was women and 51% resided in urban communities (Table 1). Fifty-eight percent of the participants were considered hypertensive and 43% had either high serum triglycerides or low HDL-C levels. Mean (±SD) dietary LA intake was 6.8 (±3.1) en% and circulating LA was 16.0 (±3.5) % total FA. Liver function markers values were available for 613 participants (97%), whose characteristics were not different from the total sample (Additional file 1**: Table S1**). The proportion of participants reporting no alcohol intake was lower in urban (48.6%) than in rural communities (66.2%), whereas proportions of participants with high alcohol intake and with obesity were similar in both communities (Additional file 1**: Table S2**).

**Table 1:** Baseline characteristics of participants of PURE-NWP-SA included in the present analyses (*n* = 633)

	*Total sample*
Age (y)	53.0 ± 10.4
Men	240 (37.9)
Body mass index (kg/m^2^)	24.9 ± 6.9
Obesity	143 (22.6)
Locality (urban)	325 (51.3)
Education level^a^	
No school or primary	247 (40.0)
Secondary or higher	371 (60.0)
Smoking status^a^	
Non-smoker	284 (44.9)
Past or current smoker	348 (55.1)
Physical activity^a^	
Low	5 (0.8)
Moderate	265 (43.4)
High	341 (55.8)
Fasting plasma glucose (mmol/L)	4.90 (4.40–5.40)
HbA1c (%)	5.60 (5.30–5.90)
Serum lipids (mmol/L)	
Total cholesterol	5.19 ± 1.31
LDL cholesterol	3.03 ± 1.13
HDL cholesterol	1.58 ± 0.64
Triglycerides	1.13 (0.84–1.69)
Liver enzymes activity (U/L)	
Gamma-glutamyl transferase	45.8 (29.7–88.0)
Alanine transaminase	17.0 (12.6–24.4)
Aspartate aminotransferase	25.0 (18.8–35.0)
HIV-positive^a^	5 (0.8)
*Dietary variables*	
Daily total energy intake (kJ)	7356 ± 3031
Available carbohydrate (en%)	58.8 ± 9.2
Protein (en%)	12.4 ± 2.3
Total fat (en%)	24.2 ± 8.7
Saturated fatty acids (en%)	5.9 ± 3.0
Monounsaturated fatty acids (en%)	6.4 ± 3.3
Polyunsaturated fatty acids (en%)	7.4 ± 3.2
n-3 fatty acids (mg/d)^a^	312 (193–474)
Trans-fatty acids (g/d)	0.18 (0.07–0.49)
Dietary cholesterol (mg/d)	149 (82–251)
Dietary fiber (g/d)	20.4 ± 9.2
Alcohol consumption^c^ (g/d)	0 (0–12.3)
No	362 (57.2)
Light	128 (20.2)
Moderate	43 (6.8)
High	100 (15.8)

Values are mean ± SD, median (Q1-Q3), or *n* (%);

*PURE-NWP-SA* Prospective Urban Rural Epidemiology North-West Province South Africa

^a^ Missing values for 15 participants for education level, 1 participant for smoking status, 22 participants for physical activity level, 1 participant for HIV status and 1 participant for n-3 fatty acids;

^b^ Available for *n* = 613;

^c^ Alcohol consumption categories: ‘No’: 0 g/d; ‘Light’: > 0 to 10 g/d (women), > 0 to 20 g/d men; ‘Moderate’: > 10–20 g/d (women), > 20–30 g/d (men); ‘High’: > 20 g/d (women), > 30 g/d (men)

Dietary LA was weakly associated with circulating LA (*r* = 0.14, *P* < 0.001). Margarine and vegetable oils, sources of dietary LA, were also associated with circulating LA with adjusted Spearman’s correlations of 0.10 (*P* = 0.011) and 0.10 (*P* = 0.009), respectively (Additional file 1**: Table S3**). Dose-response associations of circulating with dietary LA intake were evident, despite differences in sex, age or presence of obesity. Dose-response associations were observed for participants with normal plasma glucose, but not in participants with elevated plasma glucose. Within categories of similar LA intake, individuals with alcohol intake > 0 g/d had a lower mean circulating LA than individuals reporting no alcohol intake (Additional file 1**: Figure S2**).

### Dietary LA and glucose metabolism and liver function markers

Participants with higher dietary LA had on average a higher BMI, were more likely to reside in urban communities and were less likely to consume alcohol than those with lower dietary LA (Table 2). Mean (±SD) circulating LA ranged from 15.5 (±3.5) to 16.4 (±3.5) % total FA across dietary LA tertiles. Participants with higher dietary LA did not have significantly higher high-sensitivity C-reactive protein, interleukin-6 or circulating arachidonic acid than those in the lower dietary LA tertiles (Table 2).

**Table 2:** Baseline characteristics of participants across tertiles of dietary and circulating linoleic acid

	Dietary linoleic acid (en%)				Circulating linoleic acid (% total FA)			
	T1 (*n* = 211)	T2 (*n* = 211)	T3 (*n* = 211)	*P-*trend^a^	T1 (*n* = 211)	T2 (*n* = 211)	T3 (*n* = 211)	*P-*trend^a^
Median (range)	3.9 (0.4–5.3)	6.7 (5.4–8.0)	9.6 (8.0–18.4)		12.5 (6.0–14.4)	16.0 (14.4–17.7)	19.5 (17.7–25.7)	
Age (y)	52.4 ± 9.0	52.8 ± 11.3	53.8 ± 10.6	0.18	53.8 ± 9.4	54.2 ± 11.2	50.9 ± 10.1	0.004
Men	91 (43.1)	80 (37.9)	69 (32.7)	0.09	76 (36.0)	82 (38.9)	82 (38.9)	0.78
Body mass index (kg/m^2^)	23.8 ± 6.5	25.0 ± 7.5	25.9 ± 6.7	0.002	24.4 ± 7.1	25.2 ± 7.4	25.0 ± 6.3	0.36
Obesity	40 (19.0)	47 (22.3)	56 (26.5)	0.17	40 (19.0)	52 (24.6)	51 (24.2)	0.30
Waist circumference (cm)	79.6 ± 12.3	80.2 ± 14.3	81.3 ± 12.1	0.20	80.8 ± 13.4	80.6 ± 13.5	79.6 ± 11.9	0.36
Locality (urban)	71 (33.6)	123 (58.3)	131 (62.1)	< 0.001	107 (50.7)	113 (53.6)	105 (50.0)	0.72
Education level^b^				0.12				< 0.001
No school or primary	94 (45.6)	76 (36.4)	77 (37.9)		108 (51.9)	78 (38.2)	61 (29.6)	
Secondary or higher	112 (54.4)	133 (63.6)	126 (62.1)		100 (48.1)	126 (61.8)	145 (70.4)	
Smoking status^b^				0.008				0.010
Non-smoker	84 (40.0)	87 (41.2)	113 (53.6)		81 (38.4)	92 (43.6)	111 (52.9)	
Past or current smoker	126 (60.0)	124 (58.8)	98 (46.4)		130 (61.6)	119 (56.4)	99 (47.1)	
Physical activity^b^				< 0.001				0.006
Low	1 (0.5)	3 (1.5)	1 (0.5)		1 (0.5)	2 (1.0)	2 (1.0)	
Moderate	66 (32.4)	100 (49.0)	99 (48.8)		107 (52.4)	87 (42.4)	71 (35.2)	
High	137 (67.2)	101 (49.5)	103 (50.7)		96 (47.1)	116 (56.6)	129 (63.9)	
Serum lipids (mmol/L)								
Total cholesterol	5.10 ± 1.33	5.18 ± 1.31	5.28 ± 1.30	0.16	5.13 ± 1.31	5.34 ± 1.37	5.10 ± 1.25	0.81
LDL-C	2.92 ± 1.16	3.01 ± 1.14	3.15 ± 1.08	0.047	2.87 ± 1.08	3.15 ± 1.18	3.06 ± 1.11	0.09
HDL-C	1.60 ± 0.71	1.61 ± 0.60	1.55 ± 0.59	0.42	1.65 ± 0.72	1.60 ± 0.61	1.49 ± 0.56	0.012
Triglycerides	1.06 (0.85–1.62)	1.07 (0.82–1.69)	1.20 (0.86–1.78)	0.09	1.17 (0.86–1.85)	1.15 (0.85–1.65)	1.04 (0.80–1.53)	< 0.001
High sensitivity C-reactive protein (mg/L)	3.9 (1.3–10.0)	3.2 (1.0–9.5)	3.8 (1.2–11.4)	0.67	4.1 (1.4–14.0)	3.6 (1.5–9.2)	3.5 (0.9–8.8)	0.014
Interleukin-6 (pg/mL)	3.04 (1.58–7.29)	3.00 (0.75–4.91)	2.94 (0.75–5.54)	0.13	4.50 (1.98–8.83)	2.86 (0.76–5.12)	2.09 (0.75–4.16)	0.002
HIV-positive	2 (1.0)	0 (0)	3 (1.4)	0.38	2 (1.0)	0 (0)	3 (1.4)	0.30
*Dietary variables*^*c*^								
Total energy intake (kJ/d)	7413 ± 3265	7338 ± 2979	7319 ± 2846	0.75	7699 ± 3263	7282 ± 2920	7088 ± 2875	0.038
Carbohydrate	62.7 ± 10.7	59.3 ± 7.4	54.3 ± 7.2	< 0.001	57.8 ± 9.5	59.4 ± 9.2	59.1 ± 9.0	0.15
Protein	11.4 ± 2.3	12.8 ± 2.1	13.1 ± 2.1	< 0.001	12.0 ± 2.4	12.7 ± 2.2	12.7 ± 2.3	0.001
Total fat	16.4 ± 6.1	24.6 ± 5.3	31.7 ± 6.9	< 0.001	22.6 ± 9.0	24.0 ± 8.6	26.0 ± 8.3	< 0.001
Saturated FA	3.9 ± 2.4	6.2 ± 2.5	7.6 ± 2.9	< 0.001	5.4 ± 2.8	5.9 ± 3.0	6.5 ± 3.1	< 0.001
Monounsaturated FA	3.8 ± 2.0	6.6 ± 2.4	8.9 ± 3.1	< 0.001	5.8 ± 3.1	6.5 ± 3.4	7.0 ± 3.3	< 0.001
Polyunsaturated FA	4.3 ± 2.6	7.2 ± 1.1	10.6 ± 2.1	< 0.001	7.1 ± 3.7	7.1 ± 3.0	7.9 ± 3.0	0.013
n-3 fatty acids (mg/d)	221.1 (145.5–362.4)	325.3 (209.8–497.2)	391.5 (253.5–543.7)	< 0.001	296.0 (182.2–452.5)	319.6 (207.5–487.2)	319.9 (192.6–476.2)	0.37
Trans-FA (g/d)	0.09 (0.03–0.25)	0.20 (0.08–0.48)	0.33 (0.12–0.68)	< 0.001	0.18 (0.07–0.43)	0.17 (0.06–0.48)	0.19 (0.07–0.53)	0.71
Total fiber (g/d)	20.4 ± 9.0	21.1 ± 9.3	19.9 ± 9.1	0.55	20.3 ± 9.0	20.4 ± 8.6	20.6 ± 9.9	0.75
Dietary cholesterol (mg/d)	90 (48–159)	159 (99–268)	204 (129–307)	< 0.001	151 (78–248)	148 (90–246)	143 (81–254)	0.56
Alcohol use: yes^d^	118 (55.9)	84 (39.8)	69 (32.7)	< 0.001	117 (55.4)	88 (41.7)	66 (31.3)	< 0.001
(g/d)	4.6 (0–36.2)	0 (0–11.6)	0 (0–4.3)	< 0.001	2.9 (0–27.0)	0 (0–8.6)	0 (0–5.8)	< 0.001
*Fatty acid composition*								
Arachidonic acid (20:4n-6), %	13.5 ± 2.7	13.4 ± 2.5	13.7 ± 2.3	0.43	12.9 ± 2.9	14.1 ± 2.4	13.6 ± 2.0	0.011
Estimated D6D activity (20:3n-6/18:2n-6)	0.200 ± 0.061	0.192 ± 0.055	0.187 ± 0.058	0.016	0.234 ± 0.053	0.196 ± 0.047	0.150 ± 0.040	< 0.001

Values are mean ± SD, median (Q1-Q3), or *n* (%) unless otherwise stated;

*D6D* Delta-6-desaturase, *FA* Fatty acids, *HIV* Human immunodeficiency virus

^a^*P*-values for trend for continuous variables were obtained by assigning median value of dietary or circulating linoleic acid and this value was modelled as continuous; for categorical variables p-values from (exact) Chi-squared tests were displayed;

^b^ Missing values for 15 participants for education level, 1 participant for smoking status, 22 participants for physical activity level, 1 participant for HIV status, 1 participant for dietary n-3 fatty acids and 62 participants for interleukin-6;

^c^ Dietary variables are expressed as percentage of energy unless otherwise stated;

^d^ Alcohol use: ‘No’: alcohol intake = 0 g/d, ‘Yes’ alcohol intake > 0 g/d;

Dietary LA was not associated with plasma glucose or HbA1c after multivariable adjustments of demographic, lifestyle and dietary factors (Table 3). Participants in the highest dietary LA tertile had significantly lower geometric mean (95% CI) serum GGT than participants in the lowest tertile (T3 vs. T1: 48.8 (42.8, 55.6) vs. 65.2 (57.0, 74.5), *P-*trend = 0.008). Dietary LA was not significantly associated with serum ALT across tertiles (T3 vs. T1: 16.6 (15.2, 18.1) vs. 19.1 (17.4, 20.9), *P*-trend = 0.06). Geometric mean serum AST was lower with higher dietary LA (T3 vs. T1: 25.0 (22.8, 27.5) vs. 30.2 (27.4, 33.2), *P*-trend = 0.018). In continuous analysis per one SD, however, the associations between dietary LA and log-serum GGT, ALT or AST were not statistically significant (Table 3).

**Table 3:** Associations of dietary linoleic acid with glucose metabolism and liver function markers^a,b^

	Tertiles of dietary linoleic acid			*P-*trend^c^	β (95% CI) per 1 SD	*P*
	T1	T2	T3			
*Glucose metabolism markers*						
Plasma glucose, mmol/L						
Model 1	4.88 (4.73, 5.03)	4.84 (4.69, 4.99)	4.92 (4.77, 5.07)	0.71	0.008 (− 0.010, 0.026)	0.36
Model 2	4.90 (4.74, 5.06)	4.83 (4.68, 4.98)	4.91 (4.76, 5.06)	0.89	0.007 (− 0.013, 0.026)	0.50
Model 3	4.90 (4.72, 5.06)	4.83 (4.68, 4.98)	4.91 (4.76, 5.06)	0.93	0.010 (− 0.015, 0.036)	0.43
HbA1c (%)						
Model 1	5.61 (5.51, 5.70)	5.66 (5.56, 5.76)	5.67 (5.57, 5.77)	0.39	0.004 (−0.006, 0.014)	0.40
Model 2	5.64 (5.54, 5.74)	5.65 (5.56, 5.75)	5.65 (5.55, 5.75)	0.89	0.001 (− 0.010, 0.011)	0.92
Model 3	5.60 (5.49, 5.71)	5.65 (5.56, 5.75)	5.68 (5.57, 5.80)	0.36	0.008 (− 0.007, 0.022)	0.30
*Liver function markers (subsample, n = 613)*						
Serum GGT, U/L						
Model 1	64.8 (57.5, 73.1)	55.6 (49.3, 62.6)	50.2 (44.6, 56.7)	0.004	−0.079 (− 0.149, − 0.010)	0.025
Model 2	59.3 (52.6, 66.9)	56.4 (50.4, 63.2)	53.9 (48.0, 60.6)	0.28	−0.006 (− 0.076, 0.065)	0.88
Model 3	65.2 (57.0, 74.5)	57.0 (50.9, 63.7)	48.8 (42.8, 55.6)	0.008	−0.084 (− 0.179, 0.010)	0.08
Serum ALT, U/L						
Model 1	19.0 (17.6, 20.5)	17.2 (16.0, 18.6)	16.8 (15.6, 18.2)	0.027	−0.030 (− 0.074, 0.014)	0.18
Model 2	18.5 (17.0, 20.0)	17.4 (16.2, 18.8)	17.1 (15.8, 18.5)	0.21	−0.005 (− 0.053, 0.043)	0.84
Model 3	19.1 (17.4, 20.9)	17.4 (16.2, 18.8)	16.6 (15.2, 18.1)	0.06	−0.020 (− 0.084, 0.044)	0.54
Serum AST, U/L						
Model 1	29.0 (26.7, 31.5)	26.8 (24.6, 29.1)	26.0 (23.9, 28.3)	0.07	−0.022 (− 0.071, 0.026)	0.36
Model 2	28.3 (26.0, 30.9)	26.7 (24.6, 28.9)	26.6 (24.5, 28.9)	0.33	0.004 (−0.047, 0.055)	0.88
Model 3	30.2 (27.4, 33.2)	26.7 (24.6, 28.9)	25.0 (22.8, 27.5)	0.018	−0.041 (− 0.109, 0.027)	0.24

Values for outcome variables in dietary linoleic acid tertiles are geometric means and 95% CI. Values for β (95% CI) are natural log-transformed values;

*ALT* Alanine aminotransferase, *AST* Aspartate aminotransferase, *GGT* Gamma-glutamyl transferase

^a^ One standard deviation of dietary linoleic acid is 3.1 en%;

^b^ Multivariable models: Model 1: adjusted for age, sex; Model 2: model 1 plus education level, locality, smoking status, physical activity, alcohol intake, total energy intake, BMI; Model 3: model 2 plus fiber, available carbohydrates, saturated fatty acids, trans-fatty acids intake;

^c^*P*-values for trend were obtained by assigning median value of dietary linoleic acid and and this value was modelled as continuous

### Circulating LA and glucose metabolism and liver function markers

Mean (±SD) dietary LA ranged from 6.5 (±3.2) to 7.4 (±3.0) en% across circulating phospholipids LA tertiles. Participants with higher circulating LA were younger and less likely to consume alcohol. Circulating LA was inversely associated with serum triglycerides and inflammatory markers C-reactive protein and interleukin-6 (Table 2).

Circulating LA was not significantly associated with plasma glucose or HbA1c (Table 4). One SD increase in circulating LA (3.5% total FA) was associated with a 0.25 lower (95% CI: − 0.31, − 0.18; *P* < 0.001) log-serum GGT after adjustment for demographic and lifestyle factors, total energy intake and BMI (Table 4). Circulating LA was also inversely associated with log-serum ALT (β = − 0.050 (95% CI: − 0.096, − 0.003), *P* = 0.036) and AST (β = − 0.090 (95% CI: − 0.139, − 0.041), *P* < 0.001). In tertiles, circulating LA was also significantly inversely associated with serum GGT (T3 vs. T1: 42.3 (37.9, 47.2) vs. 77.2 (69.1, 86.4), *P-*trend< 0.001) and AST (T3 vs. T1: 24.8 (22.9, 26.9) vs. 30.2 (27.8, 32.8), *P-*trend< 0.001).

**Table 4:** Associations of circulating linoleic acid with glucose metabolism and liver function markers^a,b^

	Tertiles of circulating linoleic acid			*P-*trend^c^	β (95% CI) per 1 SD	*P*
	T1	T2	T3			
*Glucose metabolism markers*						
Plasma glucose, mmol/L						
Model 1	4.94 (4.79, 5.09)	4.88 (4.73, 5.03)	4.82 (4.67, 4.97)	0.28	−0.009 (− 0.027, 0.009)	0.34
Model 2	4.90 (4.75, 5.06)	4.88 (4.73, 5.03)	4.85 (4.70, 5.00)	0.62	−0.005 (− 0.024, 0.014)	0.59
HbA1c, %						
Model 1	5.59 (5.50, 5.69)	5.63 (5.54, 5.73)	5.71 (5.61, 5.81)	0.10	0.009 (−0.002, 0.019)	0.10
Model 2	5.61 (5.52, 5.71)	5.63 (5.53, 5.72)	5.69 (5.60, 5.79)	0.28	0.006 (−0.005, 0.016)	0.29
*Liver function markers (subsample, n = 613)*						
Serum GGT, U/L						
Model 1	85.5 (76.4, 95.7)	54.9 (49.1, 61.3)	38.7 (34.6, 43.3)	< 0.001	−0.32 (−0.39, − 0.26)	< 0.001
Model 2	77.2 (69.1, 86.4)	55.4 (49.8, 61.7)	42.3 (37.9, 47.2)	< 0.001	−0.25 (− 0.31, − 0.18)	< 0.001
Serum ALT, U/L						
Model 1	19.4 (18.0, 20.9)	17.3 (16.0, 18.6)	16.5 (15.3, 17.8)	0.004	−0.068 (− 0.112, − 0.024)	0.002
Model 2	18.9 (17.5, 20.4)	17.3 (16.1, 18.7)	16.9 (15.6, 18.2)	0.05	−0.050 (− 0.096, − 0.003)	0.036
Serum AST, U/L						
Model 1	31.5 (29.0, 34.3)	26.9 (24.7, 29.1)	23.9 (22.0, 25.9)	< 0.001	−0.121 (− 0.168, − 0.073)	< 0.001
Model 2	30.2 (27.8, 32.8)	26.9 (24.9, 29.1)	24.8 (22.9, 26.9)	0.001	−0.090 (− 0.139, − 0.041)	< 0.001

Values for outcome variables in circulating linoleic acid tertiles are geometric means and 95% CI. Values for β (95% CI) are natural log-transformed values;

*ALT* Alanine aminotransferase, *AST* Aspartate aminotransferase, *GGT* gamma-glutamyl transferase

^a^ One standard deviation of circulating linoleic acid is 3.5% total fatty acids;

^b^ Multivariable models: Model 1: adjusted for age, sex; Model 2: model 1 plus education level, locality, smoking status, physical activity, alcohol intake, total energy intake, BMI;

^c^ P-values for trend were obtained by assigning median value of circulating linoleic acid and this value was modelled as continuous;

Associations of circulating LA with serum GGT were modified by alcohol use (*P* < 0.001) and locality (*P* < 0.001). Stronger inverse associations of circulating LA with GGT were observed for participants who reported any alcohol consumption compared to those who reported no consumption, and for participants residing in the urban compared to those in the rural communities (Additional file 1**: Table S4**). Similar effect modification by locality was observed for associations of circulating LA with serum ALT (*P* = 0.006) and AST (*P* = 0.001).

### Sensitivity analysis

Excluding participants reporting high alcohol intake did not appreciably change the results (Additional file 1**: Table S5**). When participants with an indication of alcohol abuse were excluded, associations of circulating LA with serum GGT, ALT and AST were attenuated (Additional file 1**: Table S6**). Exclusion of participants with elevated plasma glucose levels resulted in a significant inverse association of dietary LA with log-serum GGT; the β (95% CI) per one SD was − 0.105 (− 0.204, − 0.006; *P* = 0.038) after exclusion as compared to − 0.084 (− 0.179, 0.010; *P* = 0.08) before exclusion (Additional file 1**: Table S7**).

## Discussion

In this cross-sectional study in 633 black South African men and women, dietary and circulating LA were not significantly associated with plasma glucose or HbA1c. However, both higher circulating and dietary LA were associated with lower levels of GGT. One SD higher circulating LA was associated with a 22% lower serum GGT and participants in the highest tertile of dietary LA had the lowest levels of serum GGT, a marker of liver function that is associated with insulin resistance and hepatic fat accumulation.

Only a few studies have reported on the relation of dietary and circulating LA with markers of glucose metabolism. As LA is the predominant type of dietary PUFA in many populations, including Africans [30], comparison of the results of the current study was also made with other studies using data on intake of n-6 or PUFA. The findings of no associations between dietary LA and glucose metabolism markers in the present study are in line with findings from previous studies in Caucasians. Dietary PUFA (mainly from plant source) was not associated with fasting glucose or HbA1c in 5675 Dutch men and women aged 45–65 years without diabetes [31]. Analyses of baseline data of two different trials in healthy middle-aged adults in Finland [32] and adults with metabolic syndrome in Spain [33] also showed no association of PUFA or LA intake with risk of impaired glucose metabolism. Two recent systematic reviews and meta-analyses of randomized controlled trials concluded that dietary n-6 or PUFA had some effect on insulin concentration but little or no effect on fasting glucose [11, 34], also when plant-based PUFA replaced saturated fatty acids intake in populations without diabetes [11].

As with dietary LA, circulating LA was not associated with glucose metabolism markers in the present study. In contrast, the investigators of the Hoorn study of 667 Dutch Caucasian participants reported a weak inverse association between circulating LA in serum and plasma glucose. However, no association between circulating LA and HbA1c was observed in the Hoorn study [35], consistent with findings of the present study. In the Hoorn study, about half of the participants had impaired glucose metabolism [35], while in this current study only 9% had elevated plasma glucose. In normal glucose metabolism plasma glucose is under tight regulation [36], which may explain null findings in the current study.

For dietary LA, generally either no or weak associations with liver enzymes were observed in the present study. However, circulating LA was inversely associated with serum liver enzymes, most notably serum GGT. Concentrations of liver enzyme GGT have previously been associated with hepatic fat accumulation and insulin resistance [37, 38]. In the Coronary Artery Risk Development in Young Adults study (44% black participants), mean dietary PUFA intake at baseline and year 7 tended also to be inversely related to serum GGT in year 10 of follow-up, although not statistically significant. However, there was no information on whether this association differed in the subgroup of black participants [39]. Results of a 10-week randomized controlled trial in Swedish individuals (15% had diabetes) with abdominal obesity showed that intervention with a diet high in n-6 PUFA (14 en% from LA) resulted in lower liver fat as compared to an isocaloric diet high in saturated fatty acids [40]. In the present study, exclusion of participants with elevated plasma glucose resulted in a somewhat stronger inverse association of dietary LA with serum GGT.

No other study seems to have specifically examined the association of circulating LA and serum GGT, but there is some evidence that circulating LA could be associated with liver fat accumulation [9, 41, 42]. First, in the European Prospective Investigation into Cancer and Nutrition-Potsdam study, higher delta-6-desaturase activity, resulting in lower circulating LA, was associated with higher serum GGT [41]. The authors suggested that the association of delta-6-desaturase activity with T2D risk might be mediated by liver fat accumulation. Second, higher circulating LA was associated with lower visceral adipose tissue in a study of 287 elderly subjects, and also with lower fat in a subgroup of 73 of these subjects [9]. In another study in 24 overweight men, circulating LA was moderately inversely correlated with visceral fat thickness [42]. A possible mechanism relating LA to liver function is through its influence on de novo lipogenesis by attenuating effects of insulin on FA synthase activity and expression [43]. Another possible mechanism may be related to oxidative stress, as elevated serum GGT is a marker of high oxidative stress [44].

A weak association between dietary and circulating LA in the present study may partly explain the present study finding that dietary LA was weaker associated with GGT than circulating LA. It is possible that alcohol intake plays a role here. Previously, in a cohort of Dutch post-myocardial infarction patients, weak correlations of dietary with circulating LA and lower circulating LA with higher alcohol intakes at similar intakes of LA [45] were reported, which were also observed in the present study. High alcohol intake is an established cause of liver fat accumulation [46] and higher alcohol intake has been associated with lower circulating LA in several cross-sectional studies [47–49]. It is, therefore, possible that the inverse association between circulating LA and GGT in this study may reflect alcohol influence. However, when participants whose liver enzyme values indicated alcohol abuse were excluded, the associations of LA and serum GGT were somewhat attenuated but remained significant. This suggests that the observed association cannot be entirely explained by alcohol.

The stronger associations between LA and GGT in urban than in rural areas is difficult to explain. A possible explanation might be related to alcohol use in the current study population. The urbanization process in South Africa has been linked to a change in alcohol consumption pattern [50]. A difference in obesity status is not a likely explanation because average BMI and proportion of individuals with obesity in urban and rural communities were similar.

### Study strengths and limitations

A novel aspect of the present study is the investigation on the associations of LA with glucose metabolism and liver function in a black South African population, while most other studies investigated white Caucasian populations. The current finding that LA was inversely associated with serum GGT may be relevant because the Atherosclerosis Risk in Communities study [51] showed that GGT is a strong predictor of T2D risk in a black population as compared to ALT or AST.

The present study also contributes to evidence on the relation of dietary fat quality with T2D risk in South Africans. The current recommendation for South Africans is to consume 5–8% energy from n-6 PUFA [52]. Intake in the population seems to be within this range, which was confirmed by the data in the present study. However, most of the evidence underlying the South African dietary guidelines on dietary fat quality is based on studies in Caucasian populations [52]. The present study findings suggest that a higher dietary LA is also associated with better liver function in a black population, which support the current South African recommendation of dietary n-6 PUFA.

An important limitation of the present study is its cross-sectional design, which limits inferences on causal relationships. An additional limitation is that assessment of alcohol intake by QFFQ may not be accurate. The finding that the association of circulating LA with serum GGT was stronger in alcohol consumers than in non-consumers warrants further investigation using a more sophisticated tool to assess alcohol intake. Another limitation of the current study is lack of insulin data. Availability of this data would add more information on insulin resistance in this population.

## Conclusion

In this population of apparently healthy black South Africans, dietary and circulating LA were weakly correlated. Dietary LA and circulating LA were not significantly related to markers of glucose metabolism. However, dietary LA and in particular circulating LA were inversely related to serum GGT. This suggests that a low LA intake is related to an impaired liver function and may in this way affect long-term insulin resistance, which would explain the inverse relation between circulating LA and T2D risk observed in prospective cohort studies. The role of alcohol in the association between circulating LA and liver function warrants further research.

## Supplementary information

**Additional file 1 Table S1.** Characteristics of patients in the subsample of participants with available liver enzyme values. **Table S2.** Baseline characteristics of participants by locality. **Table S3.** Spearman’s correlations of selected food groups and circulating linoleic acid. **Table S4.** Results from stratified analyses for associations of circulating LA with markers of liver function. **Table S5.** Sensitivity analyses for the cross-sectional association of dietary and circulating LA with outcomes, including only participants without high alcohol intake (*n* = 533). **Table S6.** Sensitivity analyses for the cross-sectional association of dietary and circulating LA with outcomes, including only participants without indication of alcohol abuse (*n* = 540). **Table S7.** Sensitivity analyses for the cross-sectional association of dietary and circulating LA with outcomes, including only participants with normal plasma glucose (*n* = 577). **Figure S1.** Flow chart of selection of participants. **Figure S2.** Circulating LA across tertiles of LA intake in total sample (A) and in subgroups of sex (B), age (C), obesity (D), fasting plasma glucose (E) and alcohol intake (F).

## Data Availability

The data that support the findings of this study are available from PURE South Africa study investigators but restrictions apply to the availability of these data and so are not publicly available. Data are however available from the authors upon reasonable request and with permission of the Health Research Ethics Committee of North-West University.

## Abbreviations

ALT: Alanine aminotransferase
AST: Aspartate aminotransferase
FA: Fatty acids
GGT: Gamma-glutamyl transferase
HbA1c: Glycosylated hemoglobin
HIV: Human immunodeficiency virus
LA: Linoleic acid
PAI: Physical activity index
PUFA: Polyunsaturated fatty acids
PURE-NWP-SA: Prospective Urban Rural Epidemiology North-West Province South Africa
QFFQ: Quantified food-frequency questionnaire
T2D: Type 2 diabetes
