# Supplementary material for: Associations of linoleic acid with markers of glucose metabolism and liver function in South African adults
Source: Lipids Health Dis. 2020 Jun 16;19:138. doi: 10.1186/s12944-020-01318-3 (PMC7296762; doi:10.1186/s12944-020-01318-3)
Supplement: Supplementary file 1 — Additional file 1 Table S1. Characteristics of patients in the subsample of participants with available liver enzyme values. Table S2. Baseline characteristics of participants by locality. Table S3. Spearman’s correlations of selected food groups and circulating linoleic acid. Table S4. Results from stratified analyses for associations of circulating LA with markers of liver function. Table S5. Sensitivity analyses for the cross-sectional association of dietary and circulating LA with outcomes, including only participants without high alcohol intake (n = 533). Table S6. Sensitivity analyses for the cross-sectional association of dietary and circulating LA with outcomes, including only participants without indication of alcohol abuse (n = 540). Table S7. Sensitivity analyses for the cross-sectional association of dietary and circulating LA with outcomes, including only participants with normal plasma glucose (n = 577). Figure S1. Flow chart of selection of participants. Figure S2. Circulating LA across tertiles of LA intake in total sample (A) and in subgroups of sex (B), age (C), obesity (D), fasting plasma glucose (E) and alcohol intake (F). [file 12944_2020_1318_MOESM1_ESM.docx]

Additional file 1

Article : Associations of linoleic acid with markers of glucose metabolism and liver function in South African adults

Authors : Pertiwi, Küpers, Geleijnse, Zock, Wanders, Kruger, van Zyl, Kruger, Smuts

Contents :

Table S1 Characteristics of patients in the subsample of participants with available liver enzyme values

Table S2 Baseline characteristics of participants by locality

Table S3 Spearman’s correlations of selected food groups and circulating linoleic acid

Table S4 Results from stratified analyses for associations of circulating LA with markers of liver function

Table S5 Sensitivity analyses for the cross-sectional association of dietary and circulating LA with outcomes, including only participants without high alcohol intake (n=533)

Table S6 Sensitivity analyses for the cross-sectional association of dietary and circulating LA with outcomes, including only participants without indication of alcohol abuse (n=540)

Table S7 Sensitivity analyses for the cross-sectional association of dietary and circulating LA with outcomes, including only participants with normal plasma glucose (n=577)

Figure S1 Flow chart of selection of participants

Figure S2 Circulating LA across tertiles of LA intake in total sample (A) and in subgroups of sex (B), age (C), obesity (D), fasting plasma glucose (E) and alcohol intake (F)

Table S1 Characteristics of patients in the subsample of participants with available liver enzyme values^a,b^

|  | *Values for subsample of 613* |
| --- | --- |
| Age (y) | 53.0 ± 10.4 |
| Men | 232 (37.8) |
| Body mass index (kg/m^2^) | 24.8 ± 6.9 |
| Obesity | 137 (22.4) |
| Locality (urban) | 318 (51.9) |
| Education level (no school or primary)^a^ | 238 (39.8) |
| Smoking status^a^ |  |
| Non-smoker | 275 (44.9) |
| Past or current smoker | 337 (55.1) |
| Physical activity^a^ |  |
| Low | 5 (0.8) |
| Moderate | 256 (43.2) |
| High | 331 (55.9) |
| Plasma glucose (mmol/L) | 4.90 (4.40-5.40) |
| HbA1c (%) | 5.60 (5.30-5.90) |
| Serum lipids (mmol/L) |  |
| Total cholesterol | 5.19 ± 1.31 |
| LDL-C | 3.03 ± 1.13 |
| HDL-C | 1.59 ± 0.64 |
| Triglycerides | 1.13 (0.84-1.67) |
| Liver enzymes activity (U/L) |  |
| Gamma-glutamyl transferase | 45.8 (29.7-88.0) |
| Aspartate aminotransferase | 25.0 (18.8-35.0) |
| Alanine transaminase | 17.0 (12.6-24.4) |
| HIV-positive | 5 (0.8) |
| *Dietary variables* |  |
| Daily total energy intake (kJ) | 7332 ± 3005 |
| Carbohydrate (en%) | 58.7 ± 9.3 |
| Protein (en%) | 12.4 ± 2.3 |
| Total fat (en%) | 24.2 ± 8.8 |
| Saturated fatty acids (en%) | 5.9 ± 3.0 |
| Monounsaturated fatty acids (en%) | 6.5 ± 3.3 |
| Polyunsaturated fatty acids (en%) | 7.4 ± 3.3 |
| Trans-fatty acids (en%) | 0.10 (0.04-0.23) |
| Dietary cholesterol (mg/d) | 148 (81-252) |
| Total fiber (g/d) | 20.3 ± 9.1 |
| Alcohol consumption (g/d)^b^ | 0 (0-13.2) |
| No | 352 (57.4) |
| Light | 119 (19.4) |
| Moderate | 43 (7.0) |
| High | 99 (16.2) |

Values are mean ± SD, median (Q1-Q3), or *n* (%);

^a^ Missing values for 15 participants for education level, 1 participant for smoking status, 21 participants for physical activity level and 1 participant for HIV status;

^b^ Alcohol consumption categories: ‘No’: 0 g/d; ‘Light’: >0 to 10 g/d (women), >0 to 20 g/d men; ‘Moderate’: >10-20 g/d (women), >20-30 g/d (men); ‘High’: >20 g/d (women), >30 g/d (men).

Table S2 Baseline characteristics of participants by locality^a,b,c^

|  | Urban participants  (n=325) | Rural participants (n=308) |
| --- | --- | --- |
| Age (y) | 53.8 ± 10.7 | 52.1 ± 9.8 |
| Men | 136 (41.8) | 104 (33.8) |
| Body mass index (kg/m^2^) | 25.2 ± 7.1 | 24.5 ± 6.7 |
| Obesity | 74 (22.8) | 69 (22.4) |
| Locality (urban) | 325 (100) | 0 (0) |
| Education level^a^ |  |  |
| No school or primary | 89 (28.0) | 158 (52.7) |
| Secondary or higher | 229 (72.0) | 142 (47.3) |
| Smoking status^a^ |  |  |
| Non-smoker | 142 (43.8) | 142 (46.1) |
| Past or current smoker | 182 (56.2) | 166 (53.9) |
| Physical activity^a^ |  |  |
| Low | 2 (0.6) | 3 (1.0) |
| Moderate | 229 (72.0) | 36 (12.3) |
| High | 87 (27.4) | 254 (86.7) |
| Plasma glucose (mmol/L) | 5.00 (4.30-5.50) | 4.70 (4.40-5.20) |
| HbA1c (%) | 5.60 (5.30-5.90) | 5.60 (5.30-5.90) |
| Serum lipids (mmol/L) |  |  |
| Total cholesterol | 5.28 ± 1.33 | 5.09 ± 1.29 |
| LDL-C | 3.04 ± 1.11 | 3.01 ± 1.16 |
| HDL-C | 1.54 (1.18-1.97) | 1.39 (1.10-1.82) |
| Triglycerides | 1.16 (0.85-1.78) | 1.08 (0.82-1.52) |
| Liver enzymes activity (U/L)^b^ |  |  |
| Gamma-glutamyl transferase | 49.0 (32.4-97.3) | 40.7 (27.9-77.0) |
| Alanine transaminase | 17.5 (12.8-25.0) | 16.7 (12.1-23.0) |
| Aspartate aminotransferase | 26.0 (20.7-39.0) | 23.3 (17.3-32.9) |
| HIV-positive | 1 (0.3) | 4 (1.3) |
| *Dietary variables* |  |  |
| Daily total energy intake (kJ) | 8172 ± 3117 | 6496 ± 2684 |
| Available carbohydrate (en%) | 54.9 ± 7.2 | 62.9 ± 9.5 |
| Protein (en%) | 13.4 ± 2.1 | 11.4 ± 2.1 |
| Total fat (en%) | 27.8 ± 8.0 | 20.4 ± 7.8 |
| Saturated fatty acids (en%) | 7.2 ± 2.8 | 4.6 ± 2.6 |
| Monounsaturated fatty acids (en%) | 8.0 ± 3.1 | 4.8 ± 2.6 |
| Polyunsaturated fatty acids (en%) | 7.9 ± 2.8 | 6.8 ± 3.6 |
| Linoleic acid (en%) | 7.5 ± 2.8 | 6.2 ± 3.2 |
| Trans-fatty acids (g/d) | 0.43 (0.16-0.72) | 0.09 (0.04-0.18) |
| Dietary cholesterol (mg/d) | 212 (136-309) | 106 (53-165) |
| Dietary fiber (g/d) | 22.8 ± 10.1 | 18.0 ± 7.2 |
| Alcohol intake (g/d)^c^ | 1.9 (0-15.6) | 0 (0-6.0) |
| No | 158 (48.6) | 204 (66.2) |
| Light | 79 (24.3) | 49 (15.9) |
| Moderate | 34 (10.5) | 9 (2.9) |
| High | 54 (16.6) | 46 (14.9) |
| Circulating linoleic acid (% total fatty acids) | 16.0 ± 3.2 | 15.9 ± 3.7 |

Values are mean ± SD, median (Q1-Q3), or *n* (%);

^a^ Missing values for 15 participants for education level, 1 participant for smoking status, 22 participants for physical activity level and 1 participant for HIV status;

^b^ Only available for n=613;

^c^ Alcohol consumption categories: ‘No’: 0 g/d; ‘Light’: >0 to 10 g/d (women), >0 to 20 g/d men; ‘Moderate’: >10-20 g/d (women), >20-30 g/d (men); ‘High’: >20 g/d (women), >30 g/d (men);

HIV, human immunodeficiency virus.

Table S3 Spearman’s correlations (*r_S_* ) of selected food groups and circulating linoleic acid^a,b^

| Dietary components | *r_S_* | *P* |
| --- | --- | --- |
| Cereal and cereal products | -0.01 | 0.86 |
| Vegetables | 0.12 | 0.002 |
| Fruits | 0.01 | 0.73 |
| Meat, meat products and eggs | 0.11 | 0.006 |
| Fish and seafood | -0.03 | 0.49 |
| Fats and oils | 0.14 | <0.001 |
| Margarine | 0.10 | 0.011 |
| Vegetable oils | 0.10 | 0.009 |
| Dressings | 0.04 | 0.38 |
| Tallow | 0.04 | 0.32 |

^a^ Spearman correlations adjusted for age, sex and total energy intake

^b^ Food and food group intakes are expressed in grams/day.

Table S4 Results from stratified analyses for associations of circulating LA with markers of liver function^a^

|  | Serum GGT, U/L | | | Serum ALT, U/L | | | Serum AST, U/L | | |  |
| --- | --- | --- | --- | --- | --- | --- | --- | --- | --- | --- |
|  | β (95% CI) per 1 SD | *P* | *P-int* | β (95% CI) per 1 SD | *P* | *P-int* | β (95% CI) per 1 SD | *P* | *P-int* | |
| *Dietary LA* |  |  |  |  |  |  |  |  |  | |
| Sex |  |  | 0.08 |  |  | 0.53 |  |  | 0.014 | |
| Men (n=232) | -0.070 (-0.247, 0.108) | 0.44 |  | 0.018 (-0.095, 0.130) | 0.76 |  | -0.057 (-0.180, 0.066) | 0.36 |  | |
| Women (n=381) | -0.110 (-0.219, -0.0005) | 0.049 |  | -0.048 (-0.126, 0.030) | 0.23 |  | -0.039 (-0.121, 0.043) | 0.35 |  | |
| Age |  |  | 0.57 |  |  | 0.11 |  |  | 0.11 | |
| <65 y (n=530) | -0.101 (-0.205, 0.003) | 0.06 |  | -0.046 (-0.112, 0.020) | 0.17 |  | -0.049 (-0.123, 0.025) | 0.19 |  | |
| ≥65 y (n=83) | 0.162 (-0.069, 0.393) | 0.17 |  | 0.184 (-0.065, 0.434) | 0.14 |  | 0.141 (-0.042, 0.325) | 0.13 |  | |
| Locality |  |  | 0.16 |  |  | 0.15 |  |  | 0.28 | |
| Urban (n=318) | 0.069 (-0.094, 0.233) | 0.40 |  | 0.037 (-0.072, 0.146) | 0.50 |  | 0.012 (-0.090, 0.114) | 0.82 |  | |
| Rural (n=295) | -0.181 (-0.294, -0.068) | 0.002 |  | -0.060 (-0.140, 0.020) | 0.14 |  | -0.080 (-0.177, 0.018) | 0.11 |  | |
| Obesity |  |  | 0.58 |  |  | 0.62 |  |  | 0.84 | |
| Absent (n=476) | -0.119 (-0.232, -0.006) | 0.039 |  | -0.023 (-0.094, 0.049) | 0.53 |  | -0.064 (-0.145, 0.017) | 0.12 |  | |
| Present (n=137) | 0.082 (-0.077, 0.240) | 0.31 |  | -0.012 (-0.169, 0.145) | 0.88 |  | 0.071 (-0.050, 0.191) | 0.25 |  | |
| Alcohol use |  |  | 0.12 |  |  | 0.36 |  |  | 0.06 | |
| No (n=352) | -0.009 (-0.130, 0.113) | 0.89 |  | -0.009 (-0.098, 0.080) | 0.84 |  | 0.008 (-0.084, 0.100) | 0.86 |  | |
| Yes (n=261) | -0.105 (-0.257, 0.048) | 0.18 |  | -0.037 (-0.132, 0.059) | 0.45 |  | -0.085 (-0.190, 0.021) | 0.11 |  | |
| *Circulating LA* |  |  |  |  |  |  |  |  |  | |
| Sex |  |  | 0.08 |  |  | 0.65 |  |  | 0.67 | |
| Men (n=232) | -0.37 (-0.49, -0.24) | <0.001 |  | -0.059 (-0.143, 0.024) | 0.16 |  | -0.124 (-0.214, -0.034) | 0.007 |  | |
| Women (n=381) | -0.18 (-0.26, -0.11) | <0.001 |  | -0.045 (-0.100, 0.010) | 0.11 |  | -0.073 (-0.131, -0.015) | 0.014 |  | |
| Age |  |  | 0.94 |  |  | 0.79 |  |  | 0.77 | |
| <65 y (n=530) | -0.24 (-0.31, -0.16) | <0.001 |  | -0.049 (-0.097, -0.002) | 0.042 |  | -0.084 (-0.136, -0.031) | 0.002 |  | |
| ≥65 y (n=83) | -0.27 (-0.43, -0.12) | <0.001 |  | -0.045 (-0.230, 0.140) | 0.63 |  | -0.154 (-0.284, -0.024) | 0.021 |  | |
| Locality |  |  | <0.001 |  |  | 0.006 |  |  | 0.001 | |
| Urban (n=318) | -0.44 (-0.54, -0.33) | <0.001 |  | -0.132 (-0.208, -0.056) | <0.001 |  | -0.186 (-0.256, -0.117) | <0.001 |  | |
| Rural (n=295) | -0.08 (-0.17, 0.002) | 0.05 |  | 0.014 (-0.045, 0.072) | 0.64 |  | -0.009 (-0.080, 0.062) | 0.81 |  | |
| Obesity |  |  | 0.05 |  |  | 0.29 |  |  | 0.025 | |
| Absent (n=476) | -0.28 (-0.36, -0.21) | <0.001 |  | -0.067 (-0.119, -0.015) | 0.011 |  | -0.118 (-0.176, -0.060) | <0.001 |  | |
| Present (n=137) | -0.08 (-0.18, 0.03) | 0.17 |  | 0.040 (-0.068, 0.148) | 0.47 |  | 0.044 (-0.040, 0.128) | 0.30 |  | |
| Alcohol use |  |  | <0.001 |  |  | 0.38 |  |  | 0.14 | |
| No (n=352) | -0.16 (-0.24, -0.09) | <0.001 |  | -0.046 (-0.101, 0.009) | 0.10 |  | -0.072 (-0.129, -0.015) | 0.013 |  | |
| Yes (n=261) | -0.41 (-0.54, -0.29) | <0.001 |  | -0.081 (-0.164, 0.003) | 0.06 |  | -0.142 (-0.233, -0.051) | 0.002 |  | |

Values for β (95% CI) are natural log-transformed values;

^a^ For dietary LA, results were from model 3 and for circulating LA, results were from model 2 (Subjects and Methods);

ALT, alanine aminotransferase; AST, aspartate aminotransferase; GGT, gamma-glutamyl transferase; LA, linoleic acid; *P*-int, *P*-value for interaction.

Table S5 Sensitivity analyses for the cross-sectional association of dietary and circulating LA with outcomes, including only participants without high alcohol intake (n=533)^a,b^

|  | T1 | T2 | T3 | *P*-trend | β (95% CI) per 1 SD | *P* |
| --- | --- | --- | --- | --- | --- | --- |
| *Dietary LA* | *N=148* | *N=184* | *N=201* |  |  |  |
| Plasma glucose, mmol/L | 4.91 (4.70, 5.14) | 4.84 (4.68, 5.01) | 4.87 (4.69, 5.06) | 0.85 | 0.012 (-0.019, 0.042) | 0.46 |
| HbA1c, % | 5.64 (5.50, 5.79) | 5.70 (5.59, 5.80) | 5.70 (5.58, 5.82) | 0.60 | 0.008 (-0.010, 0.025) | 0.39 |
| Serum GGT, U/L | 56.4 (48.3, 65.8) | 49.8 (44.5, 55.7) | 43.4 (38.2, 49.4) | 0.025 | -0.056 (-0.161, 0.048) | 0.29 |
| Serum ALT, U/L | 18.0 (16.2, 20.1) | 16.7 (15.4, 18.0) | 16.2 (14.8, 17.7) | 0.20 | 0.005 (-0.068, 0.077) | 0.90 |
| Serum AST, U/L | 28.3 (25.3, 31.7) | 25.2 (23.2, 27.3) | 24.2 (22.1, 26.6) | 0.07 | -0.010 (-0.085, 0.066) | 0.80 |
|  |  |  |  |  |  |  |
| *Circulating LA* | *N=152* | *N=180* | *N=201* |  |  |  |
| Plasma glucose, mmol/L | 4.91 (4.73, 5.09) | 4.88 (4.72, 5.05) | 4.84 (4.69, 5.00) | 0.61 | -0.007 (-0.027, 0.013) | 0.49 |
| HbA1c, % | 5.66 (5.54, 5.77) | 5.63 (5.53, 5.74) | 5.75 (5.65, 5.85) | 0.22 | 0.007 (-0.005, 0.018) | 0.26 |
| Serum GGT, U/L | 66.9 (59.2, 75.7) | 49.7 (44.5, 55.4) | 38.1 (34.3, 42.3) | <0.001 | -0.22 (-0.28, -0.15) | <0.001 |
| Serum ALT, U/L | 18.3 (16.8, 20.0) | 16.5 (15.2, 17.9) | 16.1 (14.9, 17.4) | 0.041 | -0.053 (-0.101, -0.004) | 0.033 |
| Serum AST, U/L | 28.8 (26.3, 31.6) | 25.5 (23.5, 27.6) | 23.6 (21.8, 25.6) | 0.002 | -0.087 (-0.137, -0.037) | <0.001 |

Values for outcome variables in tertiles of dietary or circulating LA are geometric means and 95% CI. Values for β (95% CI) are natural log-transformed values.

^a^ For dietary LA, results were from model 3 and for circulating LA, results were from model 2 (Subjects and Methods);

^b^ High alcohol intake defined as drinking alcohol >20 g/d for women and >30 g/d for men (n=100). For liver enzymes outcomes, results were from a subset of 514 participants (Dietary LA T1=140, T2=180, T3=194; Circulating LA T1=144, T2=176, T3=194);

ALT, alanine aminotransferase; AST, aspartate aminotransferase; GGT, gamma-glutamyl transferase; LA, linoleic acid.

Table S6 Sensitivity analyses for the cross-sectional association of dietary and circulating LA with outcomes, including only participants without indication of alcohol abuse (n=540)^a,b^

|  | T1 | T2 | T3 | *P*-trend | β (95% CI) per 1 SD | *P* |
| --- | --- | --- | --- | --- | --- | --- |
| *Dietary LA* | *N=166* | *N=186* | *N=188* |  |  |  |
| Plasma glucose, mmol/L | 4.99 (4.78, 5.20) | 4.85 (4.69, 5.02) | 4.96 (4.77, 5.16) | 0.93 | 0.008 (-0.021, 0.038) | 0.59 |
| HbA1c, % | 5.68 (5.54, 5.81) | 5.68 (5.57, 5.78) | 5.70 (5.58, 5.83) | 0.82 | 0.007 (-0.010, 0.023) | 0.43 |
| Serum GGT, U/L | 47.4 (41.7, 54.0) | 49.3 (44.6, 54.6) | 44.9 (39.8, 50.5) | 0.54 | 0.006 (-0.083, 0.095) | 0.90 |
| Serum ALT, U/L | 17.6 (15.9, 19.4) | 17.2 (15.9, 18.6) | 16.5 (15.1, 18.1) | 0.43 | 0.013 (-0.055, 0.081) | 0.70 |
| Serum AST, U/L | 24.7 (22.4, 27.1) | 24.9 (23.1, 26.8) | 24.1 (22.1, 26.3) | 0.74 | 0.034 (-0.032, 0.099) | 0.32 |
|  |  |  |  |  |  |  |
| *Circulating LA* | *N=160* | *N=183* | *N=197* |  |  |  |
| Plasma glucose, mmol/L | 4.97 (4.79, 5.16) | 4.95 (4.79, 5.12) | 4.88 (4.72, 5.04) | 0.45 | -0.008 (-0.029, 0.013) | 0.44 |
| HbA1c, % | 5.67 (5.55, 5.79) | 5.66 (5.55, 5.76) | 5.73 (5.62, 5.83) | 0.46 | 0.005 (-0.006, 0.017) | 0.38 |
| Serum GGT, U/L | 60.2 (54.0, 67.1) | 46.9 (42.5, 51.8) | 38.9 (35.3, 42.9) | <0.001 | -0.18 (-0.24, -0.11) | <0.001 |
| Serum ALT, U/L | 18.1 (16.6, 19.7) | 16.7 (15.5, 18.1) | 16.7 (15.4, 18.0) | 0.19 | -0.031 (-0.079, 0.017) | 0.21 |
| Serum AST, U/L | 25.8 (23.8, 28.1) | 24.4 (22.7, 26.3) | 23.6 (22.0, 25.4) | 0.12 | -0.045 (-0.091, 0.001) | 0.06 |

Values for outcome variables in tertiles of dietary or circulating LA are geometric means and 95% CI. Values for β (95% CI) are natural log-transformed values.

^a^ For dietary LA, results were from model 3 and for circulating LA, results were from model 2 (Subjects and Methods);

^b^ Indication of alcohol abuse by liver enzyme values: serum GGT concentration twice higher than normal range (values >80 U/L), and AST/ALT ratio of at least 2:1 (n =93);

ALT, alanine aminotransferase; AST, aspartate aminotransferase; GGT, gamma-glutamyl transferase; LA, linoleic acid.

Table S7 Sensitivity analyses for the cross-sectional association of dietary and circulating LA with outcomes, including only participants with normal plasma glucose (n=577)^a,b^

|  | T1 | T2 | T3 | *P*-trend | β (95% CI) per 1 SD | *P* |
| --- | --- | --- | --- | --- | --- | --- |
| *Dietary LA* | *N=194* | *N=189* | *N=194* |  |  |  |
| Plasma glucose, mmol/L | 4.73 (4.61, 4.85) | 4.56 (4.46, 4.66) | 4.69 (4.57, 4.81) | 0.76 | 0.005 (-0.013, 0.023) | 0.60 |
| HbA1c, % | 5.54 (5.46, 5.62) | 5.52 (5.46, 5.59) | 5.55 (5.47, 5.63) | 0.85 | 0.005 (-0.005, 0.015) | 0.29 |
| Serum GGT, U/L | 62.2 (54.0, 71.7) | 56.9 (50.6, 64.1) | 47.2 (41.2, 54.1) | 0.014 | -0.105 (-0.204, -0.006) | 0.038 |
| Serum ALT, U/L | 19.0 (17.2, 20.9) | 17.0 (15.7, 18.4) | 16.1 (14.7, 17.7) | 0.036 | -0.035 (-0.102, 0.032) | 0.31 |
| Serum AST, U/L | 30.3 (27.4, 33.5) | 26.9 (24.7, 29.2) | 24.7 (22.4, 27.2) | 0.012 | -0.052 (-0.122, 0.019) | 0.15 |
|  |  |  |  |  |  |  |
| *Circulating LA* | *N=190* | *N=191* | *N=196* |  |  |  |
| Plasma glucose, mmol/L | 4.71 (4.61, 4.81) | 4.64 (4.54, 4.74) | 4.63 (4.53, 4.73) | 0.32 | -0.005 (-0.018, 0.008) | 0.44 |
| HbA1c, % | 5.52 (5.45, 5.58) | 5.51 (5.45, 5.58) | 5.58 (5.52, 5.65) | 0.16 | 0.005 (-0.002, 0.012) | 0.13 |
| Serum GGT, U/L | 75.8 (67.3, 85.3) | 54.0 (48.2, 60.4) | 41.1 (36.7, 46.1) | <0.001 | -0.24 (-0.31, -0.17) | <0.001 |
| Serum ALT, U/L | 18.3 (16.8, 19.9) | 17.0 (15.7, 18.4) | 16.6 (15.4, 18.0) | 0.12 | -0.040 (-0.088, 0.009) | 0.11 |
| Serum AST, U/L | 30.0 (27.5, 32.7) | 26.9 (24.7, 29.2) | 25.0 (23.0, 27.1) | 0.004 | -0.090 (-0.140, -0.040) | <0.001 |

Values for outcome variables in tertiles of dietary or circulating LA are geometric means and 95% confidence intervals. Values for β (SE) are natural log-transformed values.

^a^ For dietary LA, results were from model 3 and for circulating LA, results were from model 2 (Subjects and Methods);

^b^ Normal plasma glucose was defined as fasting plasma glucose <6.1 mmol/L (n=56). For liver enzymes outcomes, results were from a subset of 558 participants (Dietary LA T1=186, T2=184, T3=188; Circulating LA T1=182, T2=187, T3=189);

ALT, alanine aminotransferase; AST, aspartate aminotransferase; GGT, gamma-glutamyl transferase; LA, linoleic acid.

n=2010

Attended baseline measurements

Missing dietary fatty acids data at baseline, n=1299

Extreme energy intake at baseline, n=44

n=667

Missing plasma FA at baseline, n=0

n=667

Missing or unreliable fasting plasma glucose at baseline, n=20

Missing HbA1c, n=1

n=646

Using diabetes medication at baseline, n=13

(Among these, n=11 self-reported having diabetes)

n=633

(subsample with liver markers data, n= 613)

Figure S1 Flow chart of selection of participants


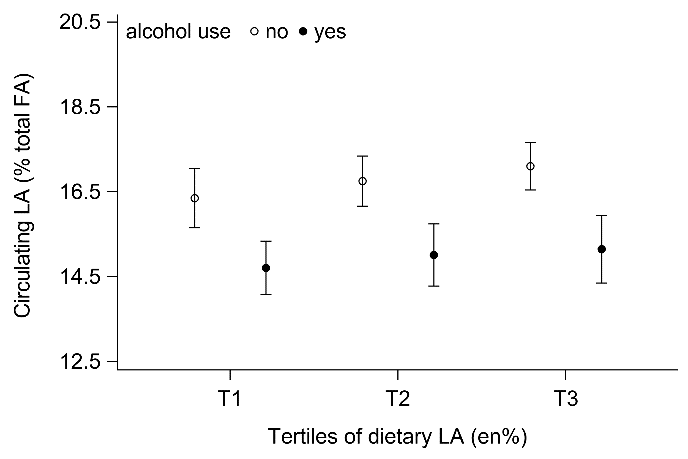

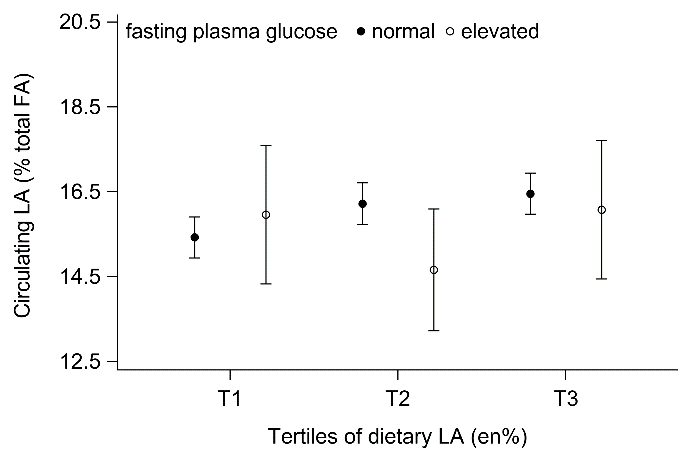

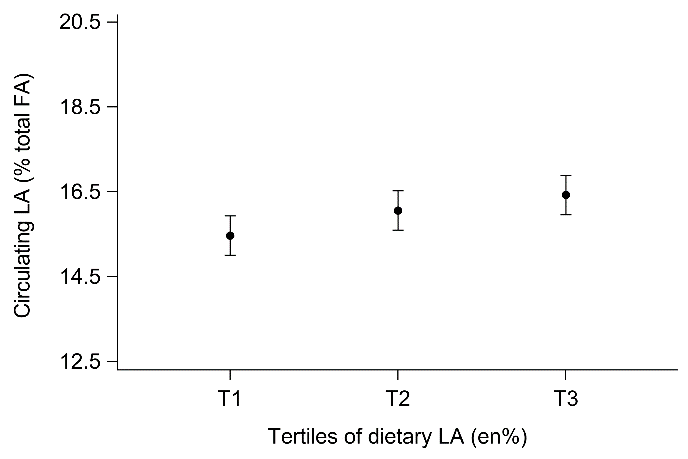

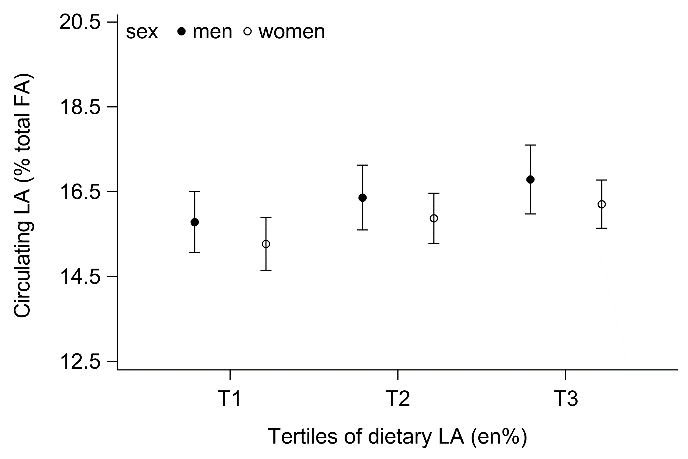

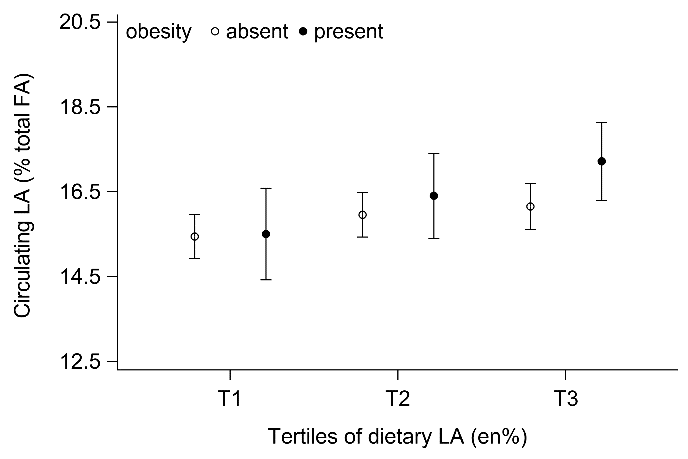

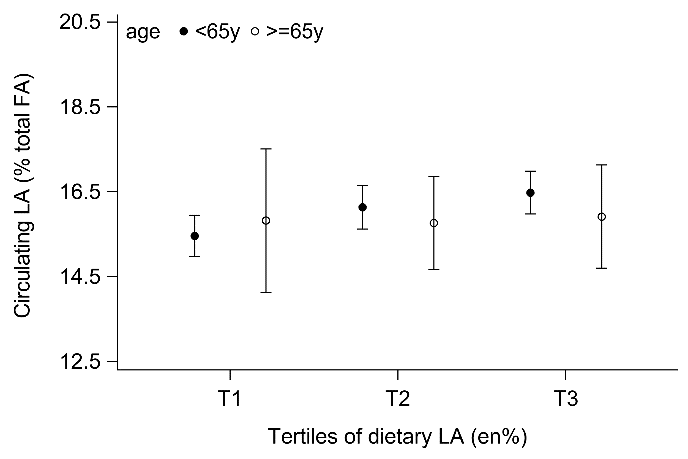


**F**

**E**

**A**

**B**

**C**

**D**

Figure S2 Circulating LA across tertiles of LA intake in total sample (A) and in subgroups of sex (B), age (C), obesity (D), fasting plasma glucose (E) and alcohol use (F)^a,b,c,d^

^a^ Values are least-squares means with 95% confidence interval, adjusted for age, sex, total energy intake;

^b^ Obesity was defined as ‘present’ when BMI ≥30.0 kg/m^2^ and ‘absent’ when BMI <30 kg/m^2^;

^c^ Alcohol use: ‘no’ included participants with alcohol intake of 0 g/d, ‘yes’ included alcohol intake >0 g/d;

^d^ Fasting plasma glucose categories: ‘elevated’ was defined as fasting plasma glucose ≥6.1 mmol/L, ‘normal’ was defined as plasma glucose <6.1 mmol/L;

LA, linoleic acid.
